# Physical Activity Level and Sedentary Behaviors among Public School Children in Dakar (Senegal) Measured by PAQ-C and Accelerometer: Preliminary Results

**DOI:** 10.3390/ijerph13100998

**Published:** 2016-10-10

**Authors:** Adama Diouf, Mbeugué Thiam, Nicole Idohou-Dossou, Ousmane Diongue, Ndé Mégné, Khady Diallo, Pape Malick Sembène, Salimata Wade

**Affiliations:** 1 Laboratoire de Nutrition, Département de Biologie Animale, Faculté des Sciences et Techniques, Université Cheikh Anta Diop (UCAD) De Dakar, Dakar-Fann BP 5005, Senegal; mbeugu@gmail.com (M.T.); nicole.dossou@ucad.edu.sn (N.I.-D.); diongue86ouz@hotmail.fr (O.D.); stecy_023@yahoo.fr (N.M.); salimata.wade@ucad.edu.sn (S.W.); 2 Division du Contrôle Médical Scolaire, Ministère de l’Education Nationale, Camp Jeremy Avenue Cheikh Anta Diop De Dakar, Dakar BP 35453, Senegal; khadydiallo@hotmail.com (K.D.); msembene@refer.sn (P.M.S.)

**Keywords:** physical activity, accelerometer, PAQ-C, school children, Senegal

## Abstract

*Background*: Physical inactivity and sedentary lifestyles are major risk factors of childhood obesity. This study aimed to measure physical activity (PA) levels by accelerometer and Physical Activity Questionnaire for Older Children (PAQ-C) among Senegalese school children and the relation with Body Mass Index (BMI) and body composition. *Methodology*: 156 pupils 8–11 years old were randomly selected in four elementary public schools of Dakar. BMI *z*-score was used to categorize children according to their weight status. PA was measured by PAQ-C in the 156 pupils and by accelerometer (Actigraph GT3X+, Pensacola, FL, USA) in a subsample of 42 children. Body composition was determined by deuterium dilution method. *Results*: PAQ-C results were comparable in the 156 and 42 pupils. The 42 pupils presented a light activity measured by accelerometer, while PAQ-C classified the majority of them (57%; *n* = 24) in the moderate PA level. Children spent most of their time (min/day) in sedentary activities and light activities than in moderate and intense activity levels. Accumulation of 60 min/day Moderate-to-Vigorous Physical Activity (MVPA) was achieved by 54.8% (*n* = 23) of the pupils. MVPA decreased in girls in relation to their body fatness. There was a significant difference in MVPA between boys and girls. Similarly, overweight/obese (45 ± 16 min/day) children had lower MVPA than their normal and underweight peers (88 ± 34 and 74 ± 36 min/day, respectively; *p* = 0.004). *Conclusions*: The two methods are inconsistent for measuring light and moderate PA levels. Although PAQ-C is an uncomplicated routine method, various activities were not adapted for genuine activities in Senegalese children and therefore needs to be validated in African children.

## 1. Background

Overweight and obesity are emerging public health problems in low- and middle-income countries. Economic transition, urbanization, dietary habits, lifestyles, and lack of physical activity have been reported as being largely responsible for the increased prevalence of obesity and related health risks [1,2,3]. Childhood obesity is a serious public health challenge as overweight and obese children are likely to stay obese and might develop non-communicable diseases (NCDs) during adulthood [4]. Elimination of physical inactivity would remove between 6% to 10% of the major NCDs such as cardiovascular disease, type 2 diabetes, and breast and colon cancers, while increasing life expectancy [5]. For positive health outcomes, the World Health Organization (WHO) recommends that children and youths aged 5–17 years should accumulate at least 60 min per day of moderate-to-vigorous intensity physical activity (MVPA) [6]. The last Lancet Physical Activity Series reported that 80% of adolescent 13–15-year-olds do not meet these current physical activity recommendations [7]. These inappropriate behaviors are perceptible in most African countries and highlight the need for more physical activity surveillance data from Africa [8]. The prevalence of physical inactivity is defined as doing none or very little physical activity at work, at home, for transport, or during discretionary time. It has been reported that less than 50% of children and youths from African countries are physically active for at least 60 min a day at least three days a week [9,10]. In Senegal, few studies have investigated the physical activity level among school children or adolescents. In 2001, Bénéfice et al. using the CSA accelerometer model 7164 reported that Senegalese adolescent spent 50% of their time in sedentary activities, and most of the attending schoolgirls were less active than the non-attending school girls [11]. Recently, Djigue by using Cooper and Ruffier tests reported that overweight/obese children living in urban areas of Dakar were significantly less active than their non-obese peers [12]. However, none of these studies has addressed WHO recommendations on the practice of physical activity in school children. Furthermore, for the evaluation of physical activity level in children and adolescents, different objectives and subjective methods have been suggested, including behavioral observation, questionnaires, pedometry, accelerometry, and doubly labelled water technique [13,14,15,16,17]. Accelerometry method has been used recently in Mozambique [18], South Africa [19], Nigeria [20], and Kenya [21]. Accelerometers are electronic devices that measure the acceleration of body movement, and enable objective quantification of the frequency, duration, and intensity of physical activity. Accelerometry provides accurate physical activity data and sedentary behavior of people [22,23]. However, the method presents weaknesses that have in the past limited its use, including the inability to assess load carrying, upper body movement, and water activity. The Actigraph type accelerometers were found to be a reliable and valid criterion measure for physical activity among children and adolescents, and have been now widely recommended [24,25,26,27]. The questionnaires constitute an alternative method, less expensive and frequently used in epidemiological studies. The International Physical Activity Questionnaire (IPAQ) was developed by WHO in 1998 to facilitate the monitoring of physical activity based on a worldwide standard. It has been validated in 12 developed countries and translated into several languages (French, Spanish, Italian, and Chinese) including recently Hausa, a Nigerian local language [20,28]. The PAQ-C (Physical Activity Questionnaire for Older Children) was developed to measure physical activity among children aged 8–14 years [14]. It is a seven-day recall questionnaire developed to measure physical activity (PA) level of children. Nevertheless, using PAQ-C questionnaire in children is problematic because they may have difficulty understanding questions and recalling activities accurately. In the last 10 years, Senegal, as other sub-Saharan African countries, has made remarkable progress on the enrolment rate in primary school (96%), but there is limited evidence on objectively measure of physical activity in school-aged children. The present study aimed to provide technical preliminary data on physical activity among Senegalese public school children in urban areas of Dakar using two commonly instruments, PAQ-C, and accelerometer.

## 2. Methods

### 2.1. Study Design and Subjects

The study used a two-stage stratified sampling design. In the first stage, four elementary public schools were randomly selected from the 149 schools of the Academic Inspection of Dakar. In the second stage, 156 pupils, 8–11 years old (39 in each school) were randomly recruited. The physical activity (PA) level was measured in all 156 by PAQ-C. Then, 42 pupils (20 boys and 22 girls) were selected for accelerometer measurements. Among the 156 children, only 9 children were overweight/obese and were all included in the accelerometer measurements. The other groups (underweight and normal weight) were randomly selected within the 147 (156–159) children to get the same number of boys and girls. Inclusion criteria were children with good general health and without physical disability and psychiatric illness. In this subgroup, PA was measured both by accelerometer and PAQ-C.

### 2.2. Ethics Approval and Consent to Participate

The study was approved by the institutional ethical committee of the University Cheikh Anta Diop of Dakar (0011/2014/CER/UCAD) and authorized by the Ministry of Education. Informed parental consent and child assent were obtained from all participants before enrollment. Study objectives and testing procedures were explained to both pedagogical team and parents. All the data were collected during the full school year.

### 2.3. Anthropometry

Anthropometric measurements were performed in all the 156 children using standardized procedures. Body weight was measured without excess outer clothing to the nearest 50 g using electronic scales (Seca 869 GmbH & Co., Hamburg, Germany). Height was measured to the nearest 0.5 cm using a portable height device (Olney, MD, USA). Height-for-age *z*-scores (HAZ), and Body Mass Index for age *z*-score (BMIZ) were calculated using WHO growth standard reference for children and youth 5–19 years of age. BMIZ was used to categorize pupils as normal weight (−2 ≤ BMIZ ≥ +1), underweight (BMIZ < −2) or overweight/obese (BMIZ > +1) groups [29]. Waist circumference (WC) was measured at a level midway between the lower rib margin and iliac crest using a tape with 1 mm accuracy (Lufkin, Baltimore, MD, USA), and the value of waist/hip ratio ≥0.5 was used to determine abdominal obesity. All the measures were performed twice. The reproducibility of the measurements were assessed by using the coefficient of variation from the duplicated measurement. CV% values of less than 5% were acceptable.

#### 2.3.1. Body Composition

Body composition (fat free mass, fat mass, % body fat) was measured by deuterium dilution method using Fourier Transform Infrared Spectrometry (FTIR IR-Affinity, Shimadzu, Nakagyo-Ku, Kyoto, Japan). Fat-free mass was calculated considering the hydration factor which varies according to age and sex among children [30].

#### 2.3.2. Physical Activity (PA) Assessment

##### Accelerometer

PA was measured using a GT3X+ triaxial monitor (ActiGraph, Pensacola, FL, USA). The monitor in conjunction with the ActiLife software version 6.9.3 (ActiGraph, Pensacola, FL, USA) application, deliver objective measure of physical activity including raw acceleration. Each child wore the monitor for seven consecutive days except for water-based activities [31].

The monitor was positioned on the right mid-axilla line at the level of the iliac crest. Raw accelerometer data were recorded in 15-s epochs, and immediately upon retrieval, the data were collapsed to 60 s epochs and analyzed using Actilife software version 6.9.3 (Actigraph, Pensacola, FL, USA). Data were validated if the subject had at least four valid days of data, including one weekend day with greater than 10 h/day of wear time. The wear time was determined according to Troiano method [32]. A valid day was defined as recording at least 600 min of measured wear time between 07:00 a.m. to bedtime 22:59 [33]. Several cut-off point have been proposed to estimate PA intensity in children [24,25,26,27]. However, as reported by Trost et al., and Romanzini et al., those of Evenson provided acceptable classification accuracy for all four levels of physical activity intensity and performed well among children of all ages [23,34]. In our study, we used the cut-off of Evenson et al. to create fours PA levels expressed in count per minute (cpm): sedentary (0–100 cpm), light (101–2295 cpm), moderate (2296–4011 cpm), and vigorous (≥4012 cpm) [25]. The actigraph monitor also records the time/percentage of time spent in sedentary behaviors and the number of minutes per day spent in Moderate-to-Vigorous Physical Activity (MVPA). MVPA was analyzed to identify the children who have accumulated at least 60 min of moderate-to-vigorous intensity physical activity daily as recommended by WHO [6].

##### PAQ-C Questionnaire

After the two stages stratified sampling yelling to a sample size of 156 children, all the children were measured for PAQ-C. Then, 42 were selected among the 156 and re-measured after seven days of accelerometer data collection. The PAQ-C contains 10 items and adapted only for measuring PA level [14]. The first item is an activity checklist including several common sports, leisure activities, and games. The remaining items (2–8) assess activity during specific periods of the day, including physical education (PE) class, recess, lunch, immediately after school, evening, and the weekend, as well as two additional questions that assess overall activity patterns during the week. The ninth item concerns the frequency of performed activities (games, sports, dance, ...) each day during the week, and the item 10th focuses on child health. Each question, except the item 10th is scored using a scale that ranges from 1 to 5. The mean of the items is used to calculate the final PAQ-C summary score. PA level was classified using Kowalski scores: Light (score = 1), Moderate (score = 2–4), and Vigorous (score = 5) that indicates a higher level of activity [14].

##### Leisure-Time Sedentary Behavior

A structured questionnaire was used to collect demographic and socio-economic characteristics, sedentary lifestyles, physical activity, nutrition knowledge, attitude, and dietary practices of the child. In the sedentary lifestyles section, information on child activities during their leisure time such as television viewing, computer use, game playing, and outdoor activities was collected.

### 2.4. Statistical Analysis

Data were computed using Epidata version 3.1 (The Epidata Association, Odense, Denmark), WHO AnthroPlus version 1.04 (WHO, Geneva, Switzerland), Excel 97-2003 (Microsoft Corporation, Redmond, WA, USA) and Stata/Special 11.1 (STATA Corporation, College Station, TX, USA) Softwares. Characteristics of the children were presented as mean ± SD and percentage. Mean differences by sex were compared using independent-sample *t-*test and analysis of variance (ANOVA) associated with a post hoc test (Bonferroni) to compare children according to the weight status. Yate’s chi-square was used to compare the physical activity levels by sex and by weight status. Pearson correlation was used to assess the association between variables. *p* values < 0.05 were considered statistically significant.

## 3. Results

### 3.1. Characteristics of the Pupils

Descriptive characteristics of the children by sex and weight status are shown in Table 1. The 42 pupils comprised 20 males and 22 females. Mean age was 9.9 ± 1 years with mean BMIZ of −0.38 ± 1.3, and mean HAZ of 0.20 ± 1.14. Any significant difference related to age, BMI *z*-score and HAZ was observed between boys and girls. Stunting (HAZ < −2) affected two children (one girl and one boy). According to BMIZ, 12 children were underweight (BMIZ < −2), 21 had normal weight (BMIZ ≥ −2), and 9 were overweight/obese (BMIZ > +1). Fat free mass was significantly higher (*p* = 0.003) and % body fat lower (*p* = 0.012) in boys compared to girls. Abdominal obesity, defined by waist:hip ratio ≥ 0.5, was observed in two boys and one girl. Analysis of the sedentary behavior showed that the same lifestyles were observed in boys as in girls. Most of them (55%) spent their time watching television/or playing computer/games rather than doing outdoor playing or active team sports with their friends.

### 3.2. PA Level and Sedentary Behaviors by Accelerometer

All the 42 children were included in the accelerometer data analysis because they provided at least four valid days of wear time, and wore the Actigraph for an average of 13 ± 1 h per valid day. For the number of valid days, 26 pupils wore the accelerometer for seven consecutive days and the remaining 16 children for four to six days. The mean duration of the actigraph wearing (6 ± 1 days) was comparable between boys and girls and between weight groups (Table 2 and Table 3). Mean cpm was 1242 ± 332 (range from 721 to 2168 cpm). All the children presented light PA level according to Evenson cut-off. The children spent most of their time (min/day) in sedentary (422 ± 83) and light (302 ± 59) activities than in moderate (53 ± 20) and vigorous (21 ± 16) activities and any differences were observed by sex and weight status in the time spent in each activity intensity. Overweight/obese pupils spent less time in moderate (*p* = 0.022) and vigorous (*p* = 0.008) activities than their normal and underweight counterpart (Table 2 and Table 3). Mean MVPA were significantly lower in girls than in boys (*p* = 0.049) and in overweight/obese children (45 ± 16 min/day; *p* = 0.005) compared to their normal (88 ± 34 min/day) and underweight (74 ± 36) peers. Fifty-five percent (55%; *n* = 23) of the children (52.2% for boys and 47.8% for girls; *p* = 0.516) accumulated 60 min/day MVPA as recommended by WHO for children and youths (Table 2). This proportion was significantly lower in overweight/obese than in normal weight pupils (69% vs. 8.7%; *p* = 0.014). A significant and negative correlation was observed between MVPA and % body mass in girls (*r* = −0.56; *p* = 0.012), but not in boys.

### 3.3. PA Level by PAQ-C Questionnaire

Mean PAQ-C score of the 156 children and of the subsample of 42 pupils were comparable (2.34 ± 0.74 vs. 2.42 ± 0.85; *p* = 0.549). However, PA score was significantly higher in boys than in girls among the 156 children (2.72 ± 0.66 vs. 1.96 ± 0.62; *p* = 0.0001), as well as among the subsample of 42 pupils (2.90 ± 0.60 vs. 1.98 ± 0.75; *p* = 0.0002). Light and moderate PA levels among the 156 (35% and 65%) were similar as those observed among the subsample of 42 pupils (43% and 57%) and the respective proportions were comparable (*p* > 0.05). The PA level of the subsample of the 42 children was presented by sex (Table 2) and by weight status (Table 3). The proportion of children in light and moderate PA levels was comparable in the weight status groups. According to sex, the proportion of school children engaged in light PA (3 boys and 15 girls) and that of pupils engaged in moderate PA (17 boys and 7 girls) was significantly different (*p* = 0.001). Indeed, boys were more engaged in moderate activity than girls and inversely. However, these differences were not observed when data were analyzed according to weight status. None of the children presented an intense activity level (score = 5).

### 3.4. Physical Activity Level Comparison

The comparison the results of physical activity from accelerometer and PAQ-C shows that both methods were consistent for the sedentary activity (Table 2), but classified the children differently in terms of light or moderate PA levels. The accelerometer classified all the pupils (100%) in the light PA level according to Evenson cut-off, and indicated that children spent less time in moderate-to-vigorous activity, while PAQ-C classed 43% (*n* = 18) and 57% of them (*n* = 24) in light and moderate PA levels, respectively.

## 4. Discussion

The purpose of the study was to measure physical activity (PA) and sedentary behaviors among Senegalese children between 8 to 11 years of age attending public elementary school in urban areas of Dakar. PA level was measured both by accelerometer and PAQ-C questionnaire. The two methods were distinct despite allowing the classification of children in different levels of physical activity. The accelerometer measures the electrical pulses of each acceleration and/or deceleration over a given period of time (seconds, minutes, or hours) before transforming them into digital data expressed as counts per minute, while PAQ-C assess the general PA level using score. Few published data exist on objective measure of physical activity using accelerometer among school children in sub-Saharan Africa [18,19,20,21]. Our accelerometer data were valid and consistent with the quality control accelerometer data processing since all the children wore the actigraph for at least four days, during 10 h per valid day including at least one valid weekend day [31,32,33]. In this study, we used the cut-off proposed by Evenson developed in children aged 5–16 years including black children [25]. Children spent 65% and 28% of their time in sedentary and light PA level. This result was consistent with previous data published by Benefice et al. among Senegalese adolescent girls in Niakhar, a rural area of Senegal [11]. Predominant light PA level in children and adolescents was also reported from South Africa and Mozambique [18,19]. More than 50% of the African children reached WHO recommendations for moderate-to-vigorous-activity but spent most of their times in light and sedentary activities. Supporting the practice of more moderate and vigorous exercises should be addressed to the school children particularly to the overweight/obese pupils. Although these studies have used different cut-off developed by Puyau, Evenson, and Treuth [24,25,35], the PA level detected were comparable. By contrast, when the habitual physical activity was transformed into MVPA, there was a difference in the time spent on moderate-to-vigorous PA across the studies. This variability makes data comparisons between studies and compliance with MVPA guidelines difficult, highlighting the need to harmonize cut-off values while using acceleromter in different population. To date, there is no consensus about accelerometer cut-off, but those developed by Evenson have been highly recommended as it provided acceptable classification accuracy for all four levels of physical activity intensity, and performed well among children of all ages [25,34]. In our study, weight and sex-based differences were observed in MVPA as persistently reported in the literature. Boys spent significantly more time in MVPA than girls, and overweight/obese pupils less time than their normal and underweight peers. Yet, overall, children spent only 6.6% of their time in MVPA (75 ± 35 min/day) like in Kenyan and South African children with comparable age, even though the Kenyan and South African studies were performed in a bigger sample size than our study [19,36]. In contrast, MVPA was higher in a sample of rural Mozambican and Ugandan children including adolescents [18,37]. These differences could be explained by the age of the children, and/or their living conditions (urban vs.rural areas). The proportion of children who met the WHO guidelines was higher in the Senegalese than in the Kenyan and South African pupils [19,20,21]. The main difference between such countries lies in the level of the economic development, Kenya and South Africa are emerging countries, while Senegal is still a developing country. In this study, 90% of the children who practice at least 60 min/day of MVPA were in normal or underweight status, a small proportion of overweight/obese children met that goal. Similar results have been reported from previous studies in Senegalese, as well as in South Africa, Kenya, and Canada in children and adolescents [11,19,21,22], indicating that overweight children are significantly less likely to practice physical activity than their healthy and underweight peers. Senegalese school children reported active transport to and from school, neverthless, PAQ-C categorized the majority of them in moderate level of PA, like in Nigerian, Malaysian, and Canadian children and adolescents [38,39,40,41], indicating that PAQ-C detects mainly moderate level of PA in children whatever their country of origin. PA level determined by PAQ-C was also sex dependent; girls were more engaged in light activity than boys. Such findings are consistent with early studies [38,39,40,41,42], and could be related to the type and or intensity of activities practiced during their leisure-time. Light PA detected by accelerometer and partially by the PAQ-C among the Senegalese children could be related to the hours spent sitting classroom (5–6 h) and probably to their sedentary lifestyles during their leisure-time (watching TV, playing computer/games, etc.) as previously demonstrated by Muthuri et al. in meta-analysis among sub-Saharan school-aged children [43]. Nevertheless, this study confirmed that the two methods of PA level assessment (accelerometer and PAQ-C) classify differently school children aged 8–11 years. These results are consistent with other studies [19,44,45,46,47]. It is not surprising that PA levels provided by both instruments were different. The accelerometer directly measures the accelerations induced by body movement, and it allows it to accurately capture certain movements, such as swimming or cycling; whereas the PAQ-C was designed to detect frequency of a specific behavior and provides an estimate of PA level using a recall memory. The collected information from the PAQ-C for such measure might be influenced by the desirability to report subjective behaviors, leading to under- or overestimating the global physical activity. Also, various activities listed in the PAQ-C questionnaire (cross country skiing, ice hockey, badminton, aerobics, ringette, hockey, skateboarding, baseball, softball, etc.) were not adapted to our African context and deserve to be reconsidered. These hypotheses were confirmed by several authors who found that questionnaire tend to overestimate by 36% to 173% [48,49] or underestimate by 28% [50] PA level measured using accelerometer. PAQ-C appears to have a moderate reliability [51,52], but its validity was not well established, due to the complexity of PA and sedentary behaviors measurements and the use of an appropriate gold standard method as described by Kelly et al. in a recent published debate [53].

## 5. Conclusions

The study found considerable differences in both accelerometer and PAQ-C questionnaire to measures PA level in school-aged children. PAQ-C detect light and moderate activity level, while accelerometers indicated that children spent most time in sedentary and light than in moderate-to-vigorous activity intensity. This preliminary study shows that accelerometer and PAQ-C were not equivalent for the measurement of physical activity in children. Accelerometers provide data interpretable in terms of public health, but require close monitoring to get valid data. PAQ-C appears to be an easy-to-use and alternative tool for physical activity surveillance and monitoring, but it needs to be adapted to the environmental genuine activities of African children. Overweight/obese pupils spend more time in sedentary activities and did not meet WHO recommendations. Supporting the practice of moderate exercise at least one hour a day to prevent overweight/obesity and related chronic diseases risk among school-aged children is a key recommendation of this study, although it has some limitations including the small sample size. In addition, the study was conducted in urban public school settings, therefore, observations and conclusions cannot be generalized in all Senegalese school children, especially those in private schools. Further large scale studies are required to assess the extent of PA level among public and private school-aged children in Senegal.

## Figures and Tables

**Table 1:** Characteristics of the school-aged children.

Characteristics	All (*n* = 42)	Boys (*n* = 20)	Girls (*n* = 22)	*p*
Age (year)	9 ± 1	10 ± 1	9 ± 1	0.092
Weight (kg)	32.8 ± 9	34.1 ± 9.4	31.7 ± 8.6	0.404
Height (cm)	139.6 ± 7.7	142.1 ± 6.5	137.3 ± 8.2	0.047
BMIZ	−0.38 ± 1.50	−0.46 ± 1.73	−1.71 ± 1.31	0.741
Weight status, % (*n*)				0.677
*Underweight*	28.6 (12)	35.0 (7)	22.7 (5)	
*Normal weight*	50.0 (21)	45.0 (9)	54.6 (12)	
*Overweight/obese*	21.4 (9)	20.0 (4)	22.7 (5)	
HAZ	0.20 ± 1.14	0.05 ± 0.93	−0.44 ± 1.27	0.161
Body composition				
*Fat free mass (kg)*	22.9 ± 4.1	24.7 ± 3.8	21.1 ± 3.5	0.003
*Fat mass (kg)*	6.7 ± 5.1	5.7 ± 1.2	7.5 ±1.0	0.270
*% Fat mass*	20.6	16.5	24.2	0.012
Waist circumference (cm)	56.8 ± 7.8	57.3 ± 8.05	56.4 ± 7.7	0.692
Waist/hip ratio	0.4 ± 0.04	0.4 ± 0.04	0.4 ± 0.03	0.647
Sedentary lifestyles, % (*n*)				
*Eating frequently in front of television*	55.0 (23)	47.8 (11)	52.2 (12)	0.889
*WatchingTV/computer/games every day*	55.0 (23)	43.5 (10)	56.5 (13)	0.569
*Outdoor playing/active team sports*	33.0 (14)	64.3 (9)	35.7 (5)	0.286

BMIZ: Body Mass Index *z*-score; HAZ: Height-for-age *z*-scores; Student *t*-test means comparison boys vs. girls; chi-square: comparison between proportions. Statistical significance is expressed as *p* < 0.05.

**Table 2:** Physical activity and sedentary behaviors by sex.

Variables	Overall (*n* = 42)	Boys (*n* = 20)	Girls (*n* = 22)	*p*
Accelerometer				
Number of days accelerometer worn	6 ± 1	6 ± 1	6 ± 1	0.578
Time (min/day) spent in various intensity ^1^				
*Sedentary*	422 ± 83	428 ± 89	416 ± 79	0.645
*Light*	302 ± 59	293 ± 57	312 ± 62	0.320
*Moderate*	53 ± 20	59 ± 22	47 ± 16	0.056
*Vigorous*	21 ± 16	25 ± 18	16 ± 11	0.064
*MVPA* ^2^	75 ± 35	85 ± 40	65 ± 28	0.049
*% Met 60 min MVPA daily, (n)*	54.8 (23)	52.2 (12)	47.8 (11)	0.516
PAQ-C				
Mean score	2.42 ± 0.85	2.90 ± 0.6	1.98 ± 0.75	0.0002
% PA level, (*n*)				0.001
*Light (score =* 1*)*	43.0 (18)	15.0 (3)	68.2 (15)	
*Moderate (score =* 2–5*)*	57.0 (24)	85.0 (17)	31.8 (7)	

^1^ According to Evenson cut-off; ^2^ MVPA: Moderate-to-vigorous physical activity; PA: physical activity; Student *t*-test: mean comparison boys vs. girls; chi-square: comparison proportions by sex. Statistical significance is expressed as *p* < 0.05.

**Table 3:** Physical activity and sedentary behaviors by weight status.

Variables	Weight Status			
	Normal (*n* = 21)	Underweight (*n* = 12)	Overweight/Obese (*n* = 9)	*p*
Accelerometer				
Number of days of wearing	6 ± 1	6 ± 1	5 ± 1	0.950
Time (min/day) spent in various intensity ^1^				
*Sedentary*	416 ± 89	422 ± 83	435 ± 78	0.859
*Light*	395 ± 67	316 ± 48	303 ± 56	0.640
*Moderate*	58 ± 19 ^a^	53 ± 21 ^a^	37 ± 12 ^b,c^	0.022
*Vigorous*	26 ± 15 ^a^	20 ± 16 ^a^	8 ± 4 ^b,c^	0.008
*MVPA* ^2^	88 ± 34 ^a^	74 ± 36 ^a^	45 ± 16 ^c,d^	0.004
*% Met 60 min MVPA daily, (n)*	69.6 (16)	21.7 (5)	8.7 (2)	0.014
PAQ-C mean score	2.35 ± 0.99	2.61 ± 0.82	2.31 ± 0.49	0.645
% PA level, (*n*)				0.765
*Light (score = 1)*	47.6 (10)	41.7 (5)	33.3 (3)	0.765
*Moderate (score = 2–4)*	52.4 (11)	58.3 (7)	66.7 (6)	

^1^ According to Evenson cut-off points; ^2^ MVPA: Moderate-to vigorous physical activity; In the same line, means with similar superscripts differ at *p* < 0.01; chi-square: comparison proportions by weight status. Statistical significance is expressed as *p* < 0.05.
